# Arsenic Methylation, *GSTT1, GSTM1, GSTP1* Polymorphisms, and Skin Lesions

**DOI:** 10.1289/ehp.9152

**Published:** 2006-12-20

**Authors:** Kathleen M. McCarty, Yen-Ching Chen, Quazi Quamruzzaman, Mahmuder Rahman, Golam Mahiuddin, Yu-Mei Hsueh, Li Su, Thomas Smith, Louise Ryan, David C. Christiani

**Affiliations:** 1 Yale University School of Medicine, Epidemiology and Public Health, Division of Environmental Health Sciences, New Haven, Connecticut, USA; 2 Harvard School of Public Health, Department of Environmental Health, Boston, Massachusetts, USA; 3 Dhaka Community Hospital, Dhaka, Bangladesh; 4 School of Medicine, Department of Public Health, Taipei Medical College, Taipei, Taiwan; 5 Harvard School of Public Health, Department of Biostatistics, Boston, Massachusetts, USA

**Keywords:** arsenic methylation, DMA, GST polymorphisms, methylation ratio, MMA, primary methylation, secondary methylation

## Abstract

**Objective:**

We investigated whether primary and secondary arsenic methylation ratios were associated with skin lesions and whether *GSTT1*, *GSTP1,* and *GSTM1* polymorphisms modify these relationships.

**Methods:**

A case–control study of 600 cases and 600 controls that were frequency matched on age and sex was conducted in Pabna, Bangladesh, in 2001–2002. Individual well water, urine, and blood samples were collected. Water arsenic concentration was determined using inductively coupled plasma mass spectrometry (ICP-MS). Urinary arsenic speciation was determined using high performance liquid chromatography hydride with generator atomic absorption spectrometry and ICP-MS. Genotyping was conducted using multiplex polymerase chain reaction and TaqMan.

**Results:**

A 10-fold increase in primary methylation ratio [monomethylarsonic acid (MMA)/(arsenite + arsenate] was associated with a 1.50-fold increased risk of skin lesions (multivariate odds ratio = 1.50; 95% confidence interval, 1.00–2.26). We observed significant interaction on the multiplicative scale between *GSTT1* wildtype and secondary methylation ratio [dimethylarsinic acid/MMA; likelihood ratio test (LRT), *p* = 0.01]. No significant interactions were observed for *GSTM1* or *GSTP1* or for primary methylation ratios.

**Conclusion:**

Our findings suggest that increasing primary methylation ratios are associated with an increase in risk of arsenic-related skin lesions. The interaction between *GSTT1* wildtype and secondary methylation ratio modifies risk of skin lesions among arsenic-exposed individuals.

Arsenic exposure through drinking water is a global threat to human health. It is associated with various human cancers such as skin, lung, bladder, liver, and prostate and non-cancerous outcomes such as pigmented skin lesions, Blackfoot disease, diabetes, and hypertension. Skin lesions such as hyperkeratosis and hyperpigmentation are hallmarks of chronic arsenic exposure and develop early after exposure compared with cancerous outcomes [National Research Council (NRC) 1999]. Previous studies have established the relationship between drinking water arsenic exposure and premalignant skin lesions (Ahsan et al. 2000; NRC 1999).

Mechanisms of human arsenic metabolism and related disease susceptibility, including hyperkeratosis and hyperpigmentation, are not fully understood (Kitchin 2001). Arsenic methylation has been considered a detoxification mechanism; however, it has been suggested that the methylated urinary metabolites of arsenic, specifically mono-methylarsonous acid (MMA^III^) and dimethylarsinous acid (DMA^III^), are more toxic than their precursor arsenite (As^III^) (Del Razo et al. 2001; Kitchin 2001; Styblo et al. 2000). MMA^III^ and DMA^III^ may be responsible for some of the effects of chronic arsenic exposure (Del Razo et al. 2001). Urinary arsenic species are 10–20% inorganic arsenic, 10–15% monomethylarsenic acid (MMA), 60–80% dimethylarsenic acid (DMA), and the methylated metabolites exist in trivalent and pentavalent forms (Kitchin 2001). Urinary arsenic species have been used as biomarkers of individual methylation ability and short-term exposure status (Chen et al. 2003a, 2003b).

Differences in disease susceptibility may be due to individual variability in biotransformation of arsenic, and polymorphisms in metabolic genes may contribute to this variability (Loffredo et al. 2003; Vahter 2000). Because arsenic methylation appears to affect its toxicity, it is essential to identify factors that impact methylation capacity and to better understand risk of disease.

Glutathione *S*-transferase (GST) polymorphisms alone and in concert with environmental exposures are associated with disease outcomes. Polymorphisms in GST genes may have effects on the behavior of several enzymes involved with the maintenance of cellular glutathione (GSH) levels (Strange et al. 2000). GSTs are a superfamily of enzymes that are key in the detoxification step of phase II metabolism. One role of the GSTs is to catalyze the conjugation of reduced GSH into hydrophobic and electrophilic compounds (Strange et al. 2000), along with other phase II enzymes (Hayes and Pulford 1995). GSH and related enzymes are also involved in cellular protection against reactive oxygen species (ROS) (Hayes and Pulford 1995; Ochi et al. 1994), and it has been hypothesized that maintenance of the cellular redox state may have an important role in arsenic-related pathology (Anderson 1998; Schuliga et al. 2002).

Our goals in this study were to determine whether there are associations between primary and secondary methylation ratios and risk of skin lesions and whether these associations were modified by polymorphisms in *GSTM1, GSTT1*, and *GSTP1*.

## Materials and Methods

### Study population

This case–control study was conducted in the Pabna district of Bangladesh and has been described previously (McCarty et al. 2006). Briefly, two physicians, trained by a dermatologist in characterizing arsenic-related skin lesions, identified eligible cases from the Pabna district. Physicians were blind to the exposure status of the subjects. Cases were at least 16 years of age, with one or more type of skin lesion: diffuse/spotted melanosis, diffuse/spotted keratosis, hyperkeratosis, or leukomelanosis. Cases often had more than one type of skin lesion, so the various types of skin lesions were grouped as one outcome. Controls were clinically evaluated to be free of skin lesions by visual inspection. Controls were selected randomly in a 1:1 ratio from Pabna, age of at least 16 years, living in the same village as cases but not sharing a tube well with cases. Cases and controls were frequency matched on sex and age (± 3 years). The participation rate was 98.0%; 24 subjects from 1,224 declined to participate in the study. Of the 600 cases and 600 controls enrolled in the study during 2001–2002, 11 people were excluded from the analysis on the basis of age or well-use duration. The population is ethnically homogeneous. All study participants gave informed consent. The protocol was approved by the institutional review boards at Dhaka Community Hospital, Bangladesh, and Harvard School of Public Health.

We designed the study to investigate modifiers of the association between arsenic exposure and skin lesions, not the main effects. Previous studies have investigated the main effects of arsenic exposure through drinking water and risk of skin lesions; however, studies of effect modification are not as common, particularly among regions of low exposure (Ahsan et al. 2000; Fewtrell et al. 2005; Guha Mazumder et al. 1998; Hsueh et al. 1997; Rahman et al. 2006). Accordingly, approximately 80% of controls were selected from suspected “low-exposure” arsenic (< 50 μg/L) communities and 20% of the controls were from suspected “high exposure” (≥ 50 μg/L) areas. This distribution was chosen to represent the reported background distribution of wells in Pabna (British Geological Survey and Bangladesh Department of Public Health and Engineering 2001) and to ensure heterogeneity of exposure. Individuals determined to have arsenic exposure ≥ 50 μg/L, the Bangladesh standard, were advised to change their water source.

### Interviews and sample collection

Interview and sample collection has been described previously (McCarty et al. 2006). Briefly, trained interviewers collected information through questionnaires regarding exposure history, diet, and lifestyle factors as well as collection of toenail, urine, and individual well water samples. Data were collected on liters of water/liquid per day, diet, disease history, identification of current primary water source (tube well), years of use, and use of a previous tube well.

Water samples were analyzed by the U.S. Environmental Protection Agency (EPA) Method 200.8 (U.S. EPA 1994) with inductively coupled plasma mass spectroscopy (ICP-MS) (Environmental Laboratory Services North Syracuse, NY, USA). The method limit of detection is 1 μg As/L.

One 10-mL EDTA tube was used to collect blood samples. Blood samples were stored on ice in a cooler until they were returned to the laboratory and processed with cell lysis solution. Samples were shipped suspended in cell lysis solution for DNA extraction and genotyping at the Molecular Epidemiology Laboratory, Harvard School of Public Health.

Samples of all 10 toenails from each subject were placed in individual sealed envelopes and were analyzed at Harvard School of Public Health using ICP-MS according to a previously published protocol (Chen et al. 1999). Rigorous quality assurance and control procedures were employed, with 10% of each batch of samples being repeated. Standard reference material water (NIST 1643d, Trace Elements in Water; National Institute of Standards and Technology, Gaithersburg, MD, USA) and certified human hair reference material (CRM Hair; Shanghai Institute of Nuclear Research, Academia Sinica, China) were used to validate instrument performance and digestion methods. The net concentration was calculated by subtracting detectable laboratory blank concentrations within each batch. The reported arsenic concentrations were corrected for systemic error by normalizing the sample concentrations against the measured average daily NIST 1643d As_in_ concentration. This corrected value was used in all statistical analyses.

Spot urine samples (approximately 120 mL) were collected from each subject. Urine samples were then aliquoted into two 15-mL tubes. Within 6 hr the samples were stored in a −20°C freezer. The urine samples were analyzed at a laboratory in Taiwan at the Taipei Medical University for dimethylarsonic acid (DMA), monomethylarsonic acid (MMA), As^III^, and arsenate (As^V^). High performance liquid chromatography (HPLC) was used to separate the urine samples. A hydride generator atomic absorption spectrometer (HGAAS) was used to quantify the levels of species of inorganic arsenic, As^III^, and As^V^ and the metabolites of inorganic arsenic, MMA and DMA. Urine samples were thawed at room temperature, then dispersed by ultrasonication, and filtered through a Sep-Pack C_18_ column (Mallinckrodt, Baker Inc., Phillipsburg, NJ, USA). A HPLC system (Waters 501; Waters Assoc., Milford, MA, USA) using a Nucleosil 10SB, 100A column (Phenomenex, Torrence, CA, USA) was used to separate arsenic species in each 200-μL urine sample. The HPLC was linked to a HGAAS for quantification of levels of As^III^, As^V^, MMA, and DMA. Inductively coupled plasma mass spectrometry (ICP-MS) was also used to determine the total concentration of inorganic and organic arsenic in the urine (Hsueh et al. 1997, 2002).

### Genotyping

DNA samples were stored at −80°C until processed. We evaluated the *GSTM1* and *GSTT1* genetic polymorphisms using a multiplex PCR technique, previously described by our laboratory (Liu et al. 2001). *GSTP1* polymorphism was genotyped by the 5′ nuclease assay (TaqMan) using the ABI Prism 7900HT Sequence Detection System (Applied Biosystems, Foster City, CA, USA). The primers, probes, and reaction conditions are available upon request. Genotyping for *GSTT1* and *GSTM1* was completed for 1,062 subjects, and genotyping for *GSTP1* was completed for 1,101 subjects. Genotyping was performed by laboratory personnel blinded to case–control status, and a random 5% of the samples were repeated to validate genotyping procedures. Two authors independently reviewed all genotyping results with 100% concordance.

### Statistical analysis

Data were analyzed using SAS software (version 8.2; SAS Institute Inc., Cary, NC, USA). We calculated the population characteristics and tested the significant differences between cases and controls using the chi-square and *t*-tests where appropriate. The frequency distribution of the polymorphisms was tested among controls to ensure Hardy-Weinberg equilibrium. The co-dominant model was used for *GSTP1*.

Analysis was restricted to subjects who reported well use for a period > 6 months to minimize potential for variability in well arsenic level. Subjects were frequency matched by age and sex. These covariates were included in all regression models. Arsenic level and volume of liquid consumed per day were not combined as a dose variable, as liquid volume included juice, milk, soup, and tea in addition to water. Smoking status, betel nut use, and chewing tobacco use were dichotomous categorical variables. Primary methylation ratio was defined as the concentration of MMA/ inorganic arsenic. Secondary methylation was defined as the concentration of DMA/the concentration of MMA (Chen et al. 2003a, 2003b). Generalized additive models (GAMs) were used to allow flexibility in modeling the data by relaxing the linearity assumption (Hastie and Tibshirani, 1990). Data exploration using GAMs suggested that the log-odds of case identification varied linearly with the log of primary methylation and secondary methylation ratios. Consequently, log-transformed primary methylation ratio and secondary methylation ratio were treated as continuous variables in regression models. The log-odds of case status varied linearly with the arsenic levels of well water; consequently, untransformed arsenic concentration was treated as a continuous variable in regression models. The log-odds of case status had a quadratic relationship with body mass index (BMI). To express potential quadratic effects, two BMI terms were used: BMI centered by subtracting its median, 19.1, and the square of the centered BMI. Consolidated categories for educational status and age, were established. GAM analysis was conducted in R (version 1.8.1; http://www.r-project.org/).

Odds ratios (ORs), as an estimate of the relative risk, and corresponding 95% confidence intervals (CIs) were computed for skin lesions in relation to primary and secondary methylation ratio indices by using unconditional logistic regression analyses. All regression models included age and sex as covariates. To control for potential confounding effects, we adjusted all calculations of ORs for skin lesions for level of education, smoking status, use of betel nut, use of chewing tobacco, BMI, and previous well use. Models were adjusted for the main effects of *GSTT1*, *GSTM1*, or *GSTP1*. Likelihood ratios tests were used to determine multiplicative interaction. Separate logistic regression analyses were performed in various genotype strata to assess evidence of effect modification. Additionally, effect modification was considered using, likelihood ratio tests (LRT), comparing adjusted models with main effects for GST polymorphisms and methylation ratios and an interaction term compared with a model with the main effects only.

## Results

Demographic data are presented in Table 1. Of 592 cases, diffuse melanosis accounted for 31.7% of the cases (*n* = 377), followed by leukomelanosis (*n* = 342), spotted melanosis (*n* = 145), diffuse keratosis (*n* = 117), spotted keratosis (*n* = 73), and hyperkeratosis (*n* = 40). Subjects often had multiple types of lesions; thus specific types were not analyzed separately. Cases and controls were not significantly different in terms of age, education, BMI, or sex. Controls reported longer duration of current well use, and cases reported a higher frequency of previous well use. Cases had significantly higher well and toenail arsenic concentrations than controls. Cases and controls reported similar fluid consumption. Cases reported more frequent betel nut use; however, no significant differences were observed in years of betel nut use and number of betel nuts chewed per day. Cases reported chewing tobacco use more frequently than controls; however, there was no difference in years of chewing tobacco use. Controls reported current or former cigarette use more frequently than cases.

Cases had significantly higher total urinary arsenic, DMA, MMA, As^III^, and total inorganic arsenic concentrations than controls (Table 2). Based on the bivariate analysis, cases and controls had significantly different primary and secondary methylation ratios. The frequency of the GST polymorphisms was similar between cases and controls.

In a multivariate model, a 10-fold increase in primary methylation ratio was associated with a 1.50-fold increase in odds of skin lesions (95% CI, 1.001–2.26). We did not observe a significant association between secondary methylation ratio and risk of skin lesions (OR = 0.90; 95% CI, 0.81–1.03). The association between primary methylation ratio and skin lesions remained similar after adjustment for the *GSTT1, GSTM1,* or *GSTP1* genotypes.

Adjusted ORs for the main effects of the methylation ratios on the outcome of skin lesions, stratified by genotype are presented in Table 3. For individuals with the *GSTT1* wildtype genotype, a 10-fold increase in primary methylation ratio was associated with a 1.67 increased risk of skin lesions (95% CI, 1.06–2.64). We did not detect a significant effect modification between *GSTT1*, *GSTM1,* or *GSTP1* genotypes and primary methylation ratio and risk of lesions.

In *GSTT1* wildtype individuals, a 10-fold increase in secondary methylation ratio was associated with 0.87 odds of skin lesions compared to *GSTT1* null (OR = 0.87, 95% CI, 0.76–0.99). We did not detect a significant association between secondary methylation ratio and skin lesions for the *GSTT1* null genotype. We evaluated a genotype-methylation ratio interaction model using methylation ratio as a continuous variable and the *GSTT1* genotype and found evidence of effect modification by the *GSTT1* wildtype genotype on risk of skin lesions ( LRT *p*-value = 0.01).

We did not detect a significant association between primary or secondary methylation ratios and skin lesions for the *GSTM1* genotype or effect modification by *GSTM1* on a multiplicative scale for the association between skin lesions and primary or secondary ratios. Additionally, we did not detect a significant association between primary or secondary methylation indices and skin lesions by the *GSTP1* genotypes.

## Discussion

As defined, increasing primary [MMA/ (As^III^+As^V^)] or secondary (DMA/MMA) methylation ratios would indicate more effective methylation of arsenic metabolites (Chen et al. 2003a, 2003b; Del Razo et al. 1997; Loffredo et al. 2003).We observed an overall increase in ORs of skin lesions associated with increasing primary methylation ratio. Since the function of primary methylation is to metabolize As^III^ to MMA^III^, these results are consistent with the hypothesis that increased production of MMA^III^ is a mechanism that may contribute to the adverse health effects associated with chronic arsenic exposure. Previous studies of populations in Taiwan have reported that skin cancer, including skin lesions in one study, were associated with increased primary methylation (Hsueh et al. 1997; Yu et al. 2000). A study of bladder cancer and arsenic exposure through drinking water described similar findings, although it is noted the mechanism for arsenic exposure and skin lesions is likely to be different than for bladder cancer (Chen et al. 2003b). Two studies in Taiwan found that skin cancer, including basal cell carcinoma, squamous cell carcinoma, and premalignant skin lesions, was associated with a higher percentage of MMA in the urine of exposed individuals than in controls, and these findings were consistent with findings from a study in a population in Mexico (Chen et al. 2003a; Del Razo et al. 1997; Hsueh et al. 1997). Additionally, a recent study has reported that MMA^III^ concentration in the urine of arsenic-exposed individuals was significantly higher in individuals with skin lesions (Valenzuela et al. 2005).

An increase in secondary methylation of arsenic was associated with a decreased risk of skin lesions in our study; however, this finding was not statistically significant. Controls were found to have significantly higher mean secondary methylation ratios than cases in our study population. Previous studies have reported that a decrease in secondary methylation ratio was associated with an increased risk of skin cancer with elevated arsenic exposure (Hsueh et al. 1997; Yu et al. 2000), and lower secondary methylation ratios were associated with an increased risk for bladder cancer in an arsenic-exposed population (Chen et al. 2003b).

Phase II metabolic GST enzymes conjugate metabolic intermediates into more soluble forms, which are then excreted by the body. It may be hypothesized that when the individual lacks the enzyme activity and is exposed to a xenobiotic compound, the individual would be at a greater risk of disease (Hayes and Pulford 1995; Strange et al. 2000). However, in arsenic metabolism where the products of primary and secondary methylation (MMA^III^ and DMA^III^) are suspected to be more reactive than their metabolic precursors MMA^V^ and DMA^V^, individuals with high detoxifying ability may be at greater risk of adverse effects associated with chronic arsenic exposure through drinking water (Del Razo et al. 2001; Kitchin 2001).

The mechanism for arsenic-induced skin lesions is not known nor is arsenic metabolism in humans fully understood. It is accepted that the level of cellular GSH is central to methylation (Anderson 1998; Kitchin 2001; Sakurai et al. 2004). It has been reported that the production of MMA^V^ increases the activity of GST enzymes (Sakurai et al. 2002). Results of i*n vivo* experiments identified that a GST enzyme, GST Ω, has been found to reduce MMA^V^ to MMA^III^ and to catalyze the conjugation of cellular glutathione and MMA^III^. It is proposed this depletion of GSH may be responsible for increased accumulation levels of MMA^V^ and increased toxicity (Zakharyan et al. 2001). Although the role of GST τ is not known in arsenic metabolism, it is possible that *GSTT1* may have a similar function or that this polymorphism is in linkage disequilibrium with another polymorphism responsible for this observed effect. It was also noted by another study that the cytotoxicity of MMA^V^ is between inorganic arsenic and DMA^V^ in v79 cells (Eguchi et al. 1997); however, in the depletion of GSH by an inhibitor of GSH synthase or GSH reductase, MMA^V^ is weakly cytotoxic (Biggs et al. 1997). Further mechanistic work is needed to determine the role of GST τ in arsenic metabolism.

Although the observation that the *GSTT1* null genotype modifies the association between secondary methylation and skin lesions is somewhat unexpected, some evidence exists for its biological plausibility. DMA is the metabolite produced at the end of secondary methylation. It has been reported that DMA conjugates with GSH, and this conjugate is responsible for apoptosis after GSH depletion (Sakurai et al. 2002). A previous study of GST polymorphisms and arsenic urinary metabolites reported that subjects with the *GSTT1* null genotype had an increased percentage of DMA in their urine (Chiou et al. 1997), however, we did not find a significant difference in percentage of DMA in *GSTT1* null compared with wildtype. The activity of GST τ in those that are *GSTT1* wildtype may deplete levels of GSH earlier than those that are *GSTT1* null, so it is plausible that different stages of methylation (primary vs. secondary) are important in terms of accumulation of compounds that may be related to some of the adverse effects of chronic arsenic exposure. In a previous analysis, we reported that the *GSTT1* homozygous wildtype genotype (OR = 1.56; 95% CI, 1.10–2.19) and the *GSTP1 GG* polymorphism were associated with greater odds of skin lesions, (OR = 1.86; 95% CI, 1.15–3.00) compared with the null and *GSTP1 AA* genotypes, respectively. (McCarty KM, unpublished data)

Although several studies have reported the methylation ability and compared differences in metabolite concentrations between cases and controls, sex, or age groups, this is the first study to predict odds of skin lesions based upon methylation ratios (Chiou et al. 1997; Del Razo et al. 1997, 2001; Vahter et al. 1995). Previous studies have explored the relationship with skin cancer, including skin lesions; however, skin lesions are considered noncancerous outcomes and may have a different biologic mechanism (Chen et al. 2003a; Hsueh et al. 1997; NRC 1999; Yu et al. 2000). Methylation ability is stable within an individual and not influenced by arsenic exposure (Chiou et al. 1997; Hopenhayan-Rich et al. 1996). The results of the HPLC-HGAAS ICP-MS method are not influenced by the presence of arsenobetaine or arsenocholine in urine, which arises from the organic arsenic contributed through diet (Hsueh et al. 1997). The use of the unadjusted urinary arsenic concentration from the first morning voids is an important factor in helping to minimize the effect of fluctuations in urine concentration that were unrelated to exposure (Biggs et al. 1997). Creatinine adjustment corrects for the consumption of nonarsenic-containing fluids, which dilutes urinary arsenic, and for exercise, which increases concentration (Biggs et al. 1997). However, the food frequency questionnaire administered in this study indicated that beverages other than tea and water were not frequently consumed. Additionally, it was found in several studies that creatinine adjustment may not be necessary in population studies of environmental inorganic arsenic exposure (Hinwood et al. 2002). We did not adjust for urinary creatinine in this analysis.

We acknowledge that we were unable to distinguish and quantify the trivalent methylated metabolites in the urine (MMA^III^ and DMA^III^). The trivalent species are very sensitive and measurement must occur soon after collection, making it impractical for this study design (Valenzuela et al. 2005).

Several factors related to general health may affect methylation ability. Liver cirrhosis results in significantly less MMA and significantly more DMA in urine excretion (Geubel et al. 1998). Arsenic exposure may increase risk of diabetes or hypertension (Rahman et al. 1998, 1999). Hypertension and diabetes affect the renal system, which may alter urinary arsenic ratios. Infection and diet can influence urinary pH and possibly affect urinary ratios. Based on self-report of health status, we do not believe this would have significantly affected our results; however we acknowledge this potential limitation.

There may be some degree of bias introduced by the grouping of several types of skin lesions into one outcome category. We acknowledge that different biological mechanisms may be responsible for the various types of skin lesions. Many subjects had more than one type of skin lesion. Any bias introduced by this categorization would bias the results toward the null.

The metabolism of arsenic in human and the mechanism for arsenic-related skin lesions is unclear. The relationship between methylation capacity and skin lesions needs further investigation. The findings of this study suggest new genotype–phenotype links and additional mechanistic studies are necessary to elucidate the precise mechanisms of these interactions between *GSTT1* and arsenic methylation capacity.

## Figures and Tables

**Table 1:** Characteristics of skin-lesion cases and population-based controls in Pabna, Bangladesh.

Characteristics	Controls^a^	Cases^a^	*p*-Value
Diffuse melanosis		(377)	
Leukomelanosis		(342)	
Spotted melanosis		(145)	
Diffuse keratosis		(117)	
Spotted keratosis		(73)	
Hyperkeratosis		(40)	
Mean age (years)	33.7 ± 12.6 (597)	33.9 ± 12.7 (592)	0.78
Body mass index (kg/m^2^)	20.4 ± 3.1 (597)	20.1 ± 3.1 (592)	0.17
Percent male	60.30 (360)	60.30 (357)	0.94
Mean duration of present well use (years)	10.1 ± 9.0 (592)	8.0 ± 7.2 (592)	< 0.0001
Percent reported a previous well	2.84 (17)	7.77 (45)	0.0002
Percent ever used betel nuts	24.30 (145)	27.70 (164)	0.19
Mean years of betel nut use	10.8 ± 8.9 (143)	11.0 ± 9.5 (160)	0.84
Mean number of betel nuts chewed per day	5.6 ± 3.6 (158)	5.7 ± 3.8 (149)	0.69
Percent chew tobacco leaves	16.40 (587)	17.10 (590)	0.05
Mean years of tobacco leaves chewed	9.9 ± 9.1 (95)	10.9 ± 9.4 (95)	0.46
Percent smoke cigarettes currently	30.50 (597)	26.70 (592)	0.14
Percent ever smoked	31.00 (597)	28.70 (592)	0.37
Education level	(597)	(592)	0.0005
Percent illiterate	17.40 (104)	22.90 (136)	
Percent literate (incomplete primary education)	23.80 (142)	29.40 (174)	
Percent completed primary education	11.70 (79)	11.80 (70)	
Percent completed middle school education	31.90 (191)	23.50 (139)	
Percent completed secondary education or more	13.60 (81)	26.00 (154)	

a Values are mean ± SD except where noted; *n* values are in parentheses.

**Table 2:** Measures of arsenic exposure, biomarkers of exposure and methylation capacity, and GST genotypes.

	Controls^a^	Cases^a^	*p*-Value
Mean arsenic level of current well (μg/L)	38.0 ± 99.0 (595)	174.0 ± 265.0 (592)	< 0.0001
Mean arsenic level in nail sample (μg/g)	2.8 ± 4.1 (583)	5.9 ± 7.4 (589)	< 0.0001
Mean daily total water/liquid consumption (L)	3.8 ± 1.2 (595)	3.7 ± 1.1 (592)	0.07
Mean total urinary arsenic concentration (μg/L)	51.79 ± 171.8 (597)	147.69 ± 230.8 (592)	< 0.001
Mean inorganic arsenic concentration (μg/L)	14.2 ± 28.2 (597)	21.4 ± 39.5 (592)	0.0003
Mean percent inorganic arsenic	12.80 (597)	12.90 (592)	0.50
Mean DMA concentration (μg/L)	70.0 ± 121.3 (597)	101.0 ± 152.3 (592)	0.0001
Mean percent DMA	73.80 (597)	72.40 (592)	0.05
Mean MMA concentration (μg/L)	14.9 ± 28.5 (597)	25.4 ± 49.7 (592)	< 0.0001
Mean percent MMA	14.90 (597)	13.80 (592)	0.01
Mean As^V^ concentration (μg/L)	4.1 ± 12.6 (597)	4.3 11.3 (592)	0.69
Mean As^III^ concentration (μg/L)	10.1 ± 23.8 (597)	17.1 ± 35.2 (592)	< 0.0001
Mean primary methylation ratio	1.3 ± 1.1 (597)	1.4 ± 1.2 (592)	0.23
Mean secondary methylation ratio	7.9 ± 7.5 (597)	7.5 ± 8.5 (592)	0.32
*GSTT1*			0.37
Null (17.9%)	18.80 (112)	16.90 (100)	
Wildtype (82.12%)	81.20 (483)	83.10 (491)	
*GSTM1*			0.99
Null (41.1%)	41.00 (244)	41.80 (243)	
Wildtype (58.9%)	59.00 (351)	58.20 (348)	
*GSTP1*			0.57
*AA* (53.8%)	53.60 (314)	54.00 (317)	
*AG* (38.7 %)	40.30 (236)	37.10 (218)	
*GG* (7.5%)	6.10 (36)	8.90 (52)	

a Values are mean ± SD except where noted; *n* values are in parentheses.

**Table 3:** Adjusted odds of skin lesions associated with methylation index stratified by genotype.

Strata	Primary methylation ratio OR (95% CI)	Secondary methylation ratio OR (95% CI)
*GSTT1* null	0.72 (0.26–2.02)	1.21 (0.89–1.63)
*GSTT1* wildtype	1.67 (1.06–2.64)	0.87 (0.76–0.99)
*GSTM1* null	1.93 (0.92–4.04)	0.89 (0.73–1.09)
*GSTM1* wildtype	1.27 (0.78–2.09)	0.95 (0.81–1.11)
*GSTP1*		
*AA*	1.22 (0.69–2.14)	0.83 (0.70–0.99)
*AG*	2.02 (0.99–4.10)	0.97 (0.78–1.19)
*GG*	2.23 (0.47–10.62)	1.10 (0.66–1.84)
